# Personal Air-Quality Monitoring with Sensor-Based Wireless Internet-of-Things Electronics Embedded in Protective Face Masks

**DOI:** 10.3390/s24082601

**Published:** 2024-04-18

**Authors:** Lajos Kuglics, Attila Géczy, Karel Dusek, David Busek, Balázs Illés

**Affiliations:** 1 Department of Electronics Technology, Faculty of Electronic Engineering and Informatics, Budapest University of Technology and Economics, H-1111 Budapest, Hungary; 2 Department of Electrotechnology, Faculty of Electrical Engineering, Czech Technical University, 166 27 Prague, Czech Republic; 3 Łukasiewicz Research Network-Institute of Microelectronics and Photonics, LTCC Research Group, 02-255 Kraków, Poland

**Keywords:** face mask, air quality, sensors, embedded electronics, IoT, wearable

## Abstract

In this paper, the design and research of a sensor-based personal air-quality monitoring device are presented, which is retrofitted into different personal protective face masks. Due to its small size and low power consumption, the device can be integrated into and applied in practical urban usage. We present our research and the development of the sensor node based on a BME680-type environmental sensor cluster with a wireless IoT (Internet of Things)-capable central unit and overall low power consumption. The integration of the sensor node was investigated with traditional medical masks and a professional FFP2-type mask. The filtering efficiency after embedding was validated with a head model and a particle counter. We found that the professional mask withstood the embedding without losing the protective filtering aspect. We compared the inner and outer sensor data and investigated the temperature, pressure, humidity, and AQI (Air Quality Index) relations with possible sensor data-fusion options. The novelty is increased with the dual-sensor layout (inward and outward). It was found that efficient respiration monitoring is achievable with the device. With the analysis of the recorded data, characteristic signals were identified in an urban environment, enabling urban altimetry and urban zone detection. The results promote smart city concepts and help in endeavors related to SDGs (Sustainable Development Goals) 3 and 11.

## 1. Introduction

One of the most discussed themes of COVID-19 was the application of personal protective masks. There was a lot of discourse about the positive and potentially harmful effects of mask-wearing in both the academic press and mainstream media. Although the interest and effect of COVID-19 have primarily fallen back, the issue remains as relevant today as it was then, as masks are not only used for infectious diseases; they are used in everyday work in hospitals, clean rooms, chemical environments, cities with highly polluted air, or workplaces with high dust emissions.

Rhee et al. [1] found that passive mask use significantly increases carbon dioxide levels on the user side. The weight and size of the equipment with the sensing they used made measurements impractical in real-life wearable scenarios. Georgi et al. [2] investigated several factors when different face masks were combined with physical movement. They suggested that the health risk of long-term mask use in healthy individuals is unlikely. Myhrvold et al. [3] focused on the degradation of the academic performance of students. They conducted surveys in several schools with poor air quality before and after the ventilation system’s renovation. They used air temperature, humidity, radiant temperature asymmetry, and air velocity measurements with sensors and tracer-gas monitoring equipment. László Kajtár and Levente Herczeg [4] confirmed the findings of [3] regarding decreasing student performance. Escobedo et al. [5] designed a CO_2_ detection sensor device that can be integrated into a mask to monitor the CO_2_ concentration inside the mask in real time; it has an opto-chemical carbon-dioxide sensing membrane with color data processing and NFC-enabled smartphone connection. The phone must be brought close to the circuit, and the circuit obtains energy from the nearby NFC magnetic field to take the measurement and then sends the data back via this NFC communication. A lack of a built-in power supply leaves the data collection to the user. Merkl et al. [6] measured carbon dioxide by conducting multiple measurements in a homogeneous network structure with a wireless sensor network. Recently, Salva et al. [7] focused on personal air-quality monitoring, where temperature, humidity, particulate matter PM 2.5, carbon monoxide, nitrogen dioxide, and ozone were measured in a portable device with distinct sensors, which could last for 30–60 h of work time. However, this complexity and PM analysis is still a challenge to scale down to the form factor of wearable masks, which is also due to the downsizing limitations of, e.g., PM sensors.

A. Fois et al. [8] have designed a wearable mask with a proprietary design that provides the wearer with active protection and several sensors. It can measure temperature, humidity, and pressure inside the mask, with embedded thermal measuring in the fabric of the filter as a novelty. With appropriate detection software, potentially infected individuals can be detected. This device runs on lithium-ion batteries and communicates via Bluetooth. After use, the mask automatically disinfects (only COVID-19 is mentioned) and recharges itself when placed on the charging station.

Alexander et al. [9] have developed a mask-mountable modular system that monitors the wearer using a barometer, accelerometer, microphone, magnetometer, AQI (Air Quality Index) sensor, temperature sensor, and wireless communication (Bluetooth). Power harvesting systems can be connected to the circuit via two connectors (AC/DC); the harvested energy is stored in capacitors. Based on their previous work, some of the sensors listed above have been used to measure ballistocardiography (BCG), respiratory rate, and mask fit using user calibration. Sethumadhavan et al. designed a smart mask using an air-purifying function [10]. However, their paper lacks an actual presentation of the application and possible sensor interactions. Lazaro et al. [11] presented a Smart Mask, which integrated a heat flux sensor for fast, remote healthcare monitoring. They revealed that breathing rate, coughing, and body temperature could be measured with their approach. Mudiyanselage et al. [12] used IoT-based sensor integration to determine the life expectancy of the masks. They integrated humidity and temperature sensors into their configuration by sewing and cabling through the mask, meaning the filtering parameters were violated. Also, the sensor components were not specified. Chen [13] presented a mask where identity recognition is integrated, and NFC technology is used to help prevent the spreading of the virus. Manchanda et al. [14] presented an automated mask adjuster based on sensor fusion data for better fitting. Their implementation involved a 3D-printed shell and an Arduino-based system; however, the system had a shortcoming of being inconvenient. The system used light-dependent resistor (LDR) sensors and one temperature/humidity sensor (DHT11).

The AirPop Active mask [15] from SX Innovation LTD of Hong Kong is perhaps the only sensor-equipped mask on the market available at the time of writing this paper. The built-in AQI sensor monitors local air quality and provides feedback on respiration while maintaining filtering performance. However, the outlet valve, where the electronics are stored, might be a concern during a pandemic. Data can be synchronized with a smartphone app. The device runs on a single CR1632 battery and lasts 3–6 months, with 2 h of use per day. The type of sensors used is not disclosed.

It can be seen that although several projects are addressing the issue of sensor implementation in masks, their tasks, objectives, sensor types, and actual components are very different. Currently, there is no easy-to-use, durable, autonomous solution whose primary purpose is all-around personal data collection.

In addition to the above, several smart masks without applied sensors and data collection have been developed recently. These often omit sensors and environmental monitoring but can include built-in fans and filters, microphones, speakers, and lighting. Their purpose is the user’s comfort and individual appearance. The LG PuriCare [16] is one of the most common; it has two fans and a breathing sensor that continuously assists breathing through HEPA filters in the mask. A built-in 820 mAh lithium battery powers the device. Depending on the setting, the user can adjust the fan speed in three settings, with a single charge lasting 4–8 h. Razer’s Zephyr mask is very similar in terms of use [17,18]; it also features filters and fans without the need for environmental/breathing sensing. It has a much bolder design than its peers as it uses RGB lighting inside and outside and transparent surfaces to attract attention. However, unlike the LG PuriCare mask, this one was not antibacterial certified. Also, the mask was available during the pandemic; however, its support was discontinued later.

To sum up, there are different approaches for making personal protection equipment and environmental detectors within the frame of mask application. One possibility is where embedding requires proprietary sensing solutions, such as filter-embedded sensors [8]. However, others have a bulky construction, where wearability or mask embedding is not practical [1,2,7]. Moreover, they may require mounting or the complete redesign of the mask bulk [9,14,16,17,18]; have power limitations [5]; and have violated filtering [12], and sensors may be omitted [16,17,18] or there may be no specific description of their applications. Furthermore, it must be concluded that the best solutions integrate autonomous power [7,8,9,15,16,17]; they generally present humidity, temperature [7,8,11,12,14], and AQI [9,15] measurements for user data and environmental analysis.

The works mentioned above touch upon multi-sensor-based measurements with IoT capabilities, often pushing the focus on filtration evaluation, pandemic control, and other comfort functions. However, a comprehensive device is missing from the literature that applies a multi-sensing-based, fully portable, IoT-enabled device and investigates both sides (the outside world and the proximity of the user underneath the mask) of a functional personal protective mask—in terms of temperature, humidity, pressure, and AQI sensing—while trying to retain the filtering capabilities of the system. We proposed to investigate this configuration using commercial masks retrofitted with such a device. This paper presents the research and development leading to actual measurements and validation in an urban environment.

## 2. Materials and Methods

**Figure 1:**
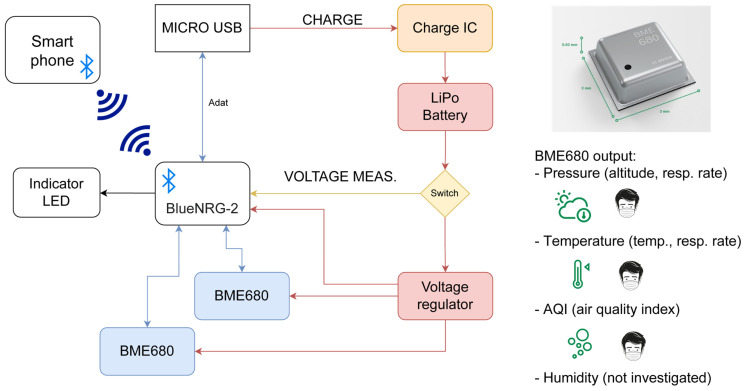
Block diagram of the electronics: the two BME680 sensors are used to monitor the outside ambience and the inside proximity to the user. The measurements are highlighted on the right side.

Figure 1 shows the concept of embedding sensors and respective electronics into two different mask types. The first type (A) was a pair of commercially available, non-branded, three-layer medical masks; the second type (B) was a commercial, but more robust, 3M Aura 9320D+ FFP2-type mask. Details about the protective masks are presented in the given subsections.

The hardware design must be small and embeddable in the form factor of face masks. The design basics were based on our previous wearable air-quality monitoring system [19] based on different sensor modules. The form factor and sensing functionality were optimized for embedding into the bulk of a mask. The block diagram and multi-sensing approach are shown in Figure 1.

The Bosch BME680 is a common, cost-effective humidity, temperature, pressure, and VOC (Volatile Organic Compound) air-quality sensor [20,21]. Its miniscule size (3 × 3 × 0.9 mm^3^) and low power consumption make it a popular choice for portable smart devices. The response time of the gas sensor (33–63% transition) is less than 1 s, and its output is the resistance value of the gas sensor. The sensor can validate the Air Quality Index (AQI), as defined in the datasheet (more information is provided in Section 2.2). The integrated signal processing electronics can communicate via I2C or SPI. Temperature, humidity, and pressure results are also directly obtainable from the digital output of the sensor. The temperature sensor embedded in the BME680 has an absolute accuracy of ±0.5 °C at ambiance and ±1 °C along the full accuracy scale (0–65 °C). The output resolution is 0.01 °C and the operating temperature range is −40 to 85 °C. The pressure sensor has the same operating temperature and full accuracy range. The maximum resolution is 0.18 Pa. The absolute accuracy is ±0.6 hPa at 300–1100 hPa, and the relative accuracy is ±0.12 hPa at 25–40 °C and 700–1100 hPa, at constant humidity. The accuracy enables altimetry. The humidity sensor has the same operating and full accuracy range, with ±3 %rh absolute accuracy at 20–80 %rH at 25 °C, 1.5 %rh hysteresis at 10→90→10 %rh at 25 °C, and 0.008 %rH resolution. Two of the sensors were designed for the circuit so that we could monitor air outside and inside the mask at the same time.

The ST Microelectronics BlueNRG-2 microcontroller SoC (System on a Chip) is a standalone, programmable BLE 5.3 (Bluetooth Low Energy)-compliant wireless microcontroller. The BlueNRG-2 has no built-in USB (Universal Serial Bus) controller, so we used an external USB-UART converter: the Silicon Labs CP2102N integrated circuit. Bluetooth LE capability is essential for designing a portable circuit. A voltage converter was applied since the BME680 has a maximum voltage of 3.6 V. We used the Texas Instruments TPS63051 buck-boost converter for this purpose. Using a switching DC-DC converter, we can generate a stable 3.3 V supply voltage regardless of the current battery operating voltage range. For battery charging, the MCP73843 charge controller IC was chosen. The single-cell Li-Ion/Li-Po battery type was chosen based on consumption estimation and format factor (Section 3.1 will present this in detail).

The final design was realized on a classic FR4 (Flame-Retardant Type 4, double-sided, 1.55 mm, 1 oz) PCB, where an SAC305-type alloy was used for soldering. A Finetech FinePlacer was used to mount the Bluetooth module. The system was reflowed using an eC-Reflow Mate (Eurocircuits, Hungary, Felsőtárkány) and SAC305 lead-free solder paste. X-ray analysis with a Nordson Dage Quadra 5 microscope was applied to investigate the surface mounting of the SoC package for the non-destructive inspection of the joints.

### 2.1. Software Design

**Figure 2:**
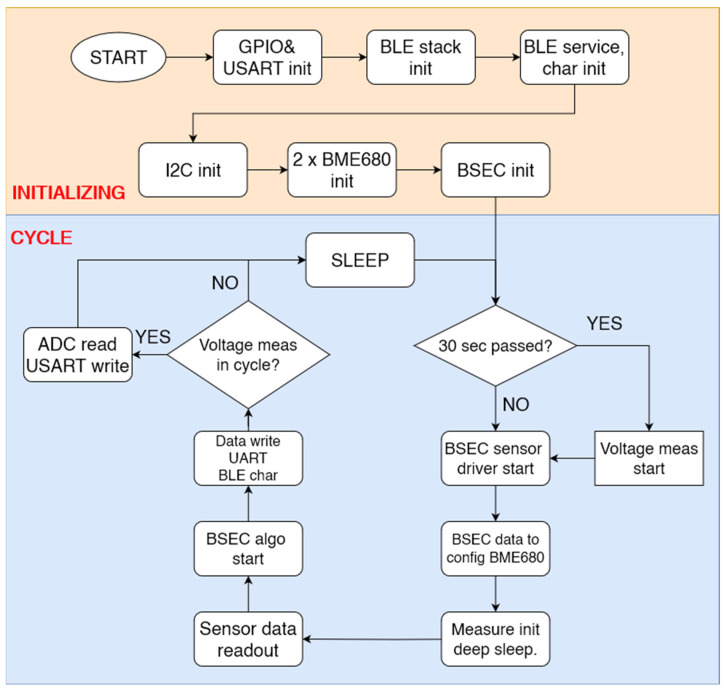
The block diagram of the embedded software.

The software design must be efficient, capable of communicating wirelessly, and work with low consumption. Figure 2 presents the block diagram of the embedded software. The software was created in the Atollic TrueStudio development environment. The program’s first step is to initialize the inputs and outputs and the communication peripherals, and then the initialization functions of the BLE stack follow. The deep sleep function is vital for low power consumption. Due to the BLE communication and the automatic calibration of the internal clock, the microcontroller can wake up to a maximum of every 15 s or even immediately after a function call. Therefore, an important part of the sleep cycle is constantly checking the time spent in sleep based on the 32 kHz counter. A string is our smallest data storage unit; a string contains the metadata needed to write/read and the data to communicate. To make data transfer as compact as possible, all the data from our two BME sensors are transmitted in a single string within a shared service; this data bundle can later be decrypted in a smartphone app. The strings operate on a subscribe-notify basis.

To communicate with the sensors, we used the BME68x API (Application Programming Interface) [22]. To use the library, we first had to write the functions that allow the API to communicate with the sensors. To initialize a sensor, we must create an array containing the sensor information, such as the sensor’s address or the pointer of the functions used to access the sensor. We then need to set the desired sampling filters and the gas sensor’s target temperature and heating time. Then, a measurement can be started at any time by identifying the appropriate function. The BSEC (Bosch Sensortec Environmental Cluster) library (Version 2.4.0.0, 2022) [23] is a software package developed by Bosch to process the data measured by the BME680 (or BME688) sensor at the measurement site, even together with data from other sensors (sensor fusion). The output is a set of compensated temperature, humidity, pressure, and AQI (Air Quality Index) values calculated from each measured characteristic. It is interesting to note that in the datasheet, IAQ is mentioned; however, this is not in terms of its general meaning (Indoor Air Quality) but as the “Index of Air Quality”. To further avoid general confusion, we use the more general AQI term throughout the paper; however, it must be noted that the AQI reference must be handled in alignment with the datasheet values and ranges [20]. The given index parameters apply only to the combination of BME680 together with the Bosch Software Environmental Cluster (BSEC) solution. According to the datasheet, the BSEC software auto-calibrates the low and high concentrations of VOC applied during initial testing to the indices of 50 and 200, respectively.

The AQI value is a relative index that can be interpreted between 0 and 500. A value of 0 indicates good air and a value of 500 indicates highly polluted air [20]. These limits are continuously variable values, and their accuracy increases with the running time of the BSEC algorithm as the various ambient air-quality conditions change. This aspect was also validated during our experiments. To reduce the memory and power requirement, only the sensor on the outside of the mask will be processed by the BSEC library for AQI detection; the inner sensor will always be in a “polluted” air state due to the exhale of the user, which is concentrated under the mask and with which the outside values would be summarized, as the masks do not filter the outside values from this aspect. Thus, the AQI value calculated from the inside of the mask would be disproportionately inaccurate in any case. During development, it was apparent that the outside sensor was not affected by the exhale significantly (while the inner sensor was saturated). To sum up, the AQI is only calculated outside, and the other parameters are all read from both sides.

After initialization, the BSEC library provides the configuration data block. Once the settings have been loaded into the sensor, a measurement is started, and then it sleeps until the data are ready. Although there is no interrupt output on the sensor to indicate exactly when to read the data, the length of the sleep can be accurately determined from the heating time of the gas sensor. After the data are read, they are passed to the BSEC algorithm, and the processed data are published in the BLE characteristics via a serial port. After that, another sleep follows, the length of which is given to us by the BSEC algorithm. At the beginning of the cycle, we always check when the last voltage measurement was taken. If it is older than 30 s, we turn on the voltage divider and start an ADC (analog-digital conversion) measurement cycle. After waking up from the first sleep and reading the sensor data, the ADC reading is read, the voltage measurement time is stored, and the voltage divider is switched off. The ADC operates with a battery voltage resolution of 6.8 mV. This is enough to give an approximate estimate of the charge.

### 2.2. Embedding the Sensors and Electronics to the Mask Structure

**Figure 3:**
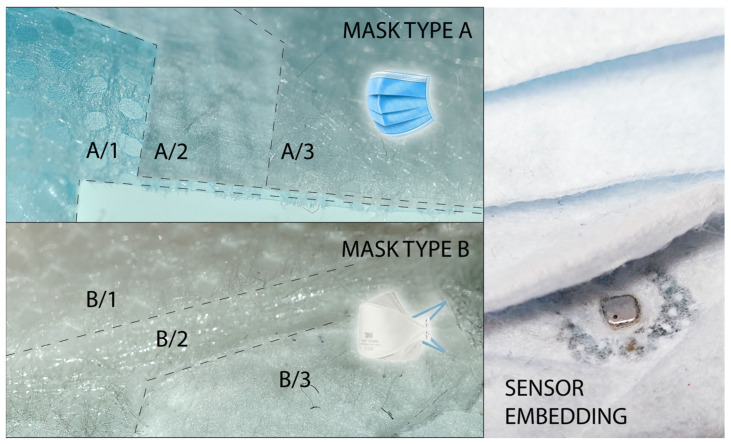
**Left**: layer structure of Mask A (**top**; A/1 inner layer, A/2 inner filter layer, A/3 outer layer) and Mask B (**bottom**; B/1—outer layer, B/2—middle, main filter layer, B/3—inner layer); **Right**: sensor embedding example into Type A (between A/1 and A/2).

For the mask embedding, we used two different types of masks. According to the literature, the commercially available, non-branded, three-layer medical mask has a supposed filtration rate that can go below 50% [24] and up to 90% [25]. This is named Mask Type A in Figure 3 and later discussions (A/1 inner layer, A/2 inner filter layer, A/3 outer layer). The layers should be composed of different polymers (such as polypropylene [25]); however, the proper composition is unknown. The 3M Aura 9320D+ FFP2 type mask, with three layers, is FFP2-certified, which means that the filter performance must be ≥94% [26], or from the other side, according to EN 149:2001 + A1:2009, the maximum filter penetration must be 6% [27]. The structure of the 3M Aura is known and is based on different layers of polypropylene filters [27] (Figure 3, B/1—outer layer, B/2—middle, main filter layer, B/3—inner layer).

The sensor-based electronics were embedded into the structure using two different approaches. Type A was very sensitive to any preparations, so two masks were adhered along the edges. Supposedly, this would increase the filtration capability. Type B was opened along the welded edge, and the electronic board and battery were positioned between the B/2 and B/3 layers. For both masks, the BME-type sensors were covered with the remaining residue-free adhesives (Kapton-type tape), and then the openings were joined and closed around the sensor with technical adhesive. After 24 h of curing, the covers were removed—this way, the sensor saturation with the departing volatiles from the adhesive was avoided. Type A and B also have different fittings; Type B has nose foam next to the nose clip and a more stable perimeter, resulting in better fitting along the face. As a technical difficulty, congested humidity inside the mask caused occasional resets in the electronics, so the bottom of the PCB was covered with Kapton-type dielectric tape to avoid further humidity precipitation.

### 2.3. Verification of the Mask Filtration Efficiency

**Figure 4:**
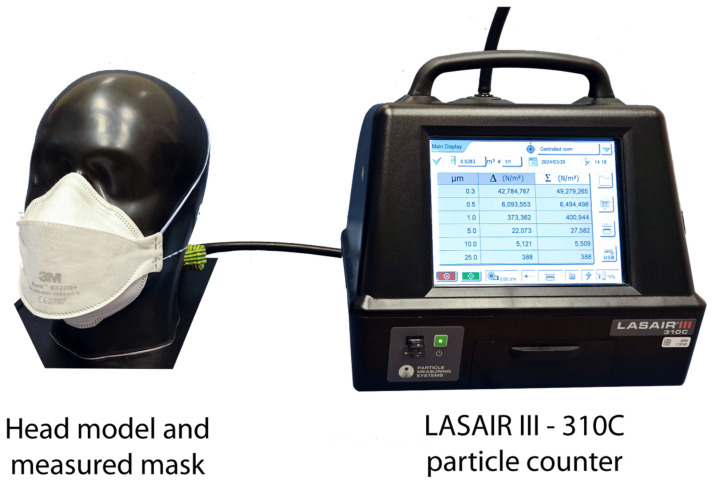
Head model with particle counter—measurement in progress for illustration purpose.

The filtration efficiency before and after the mask modification was performed using a previously developed, particle-counting-based evaluation method [28,29]. A particle counter (LASAIR III 310C) and a prepared head model (Caucasian type, made by TSI) were used for measurement, and the sample holder head with mouth flow channel was connected to the particle counter with a pipe. This way, the system could validate the mask filtration capability before and after the sensor insertion. The setup is shown in Figure 4.

The portable aerosol particle counter, Lasair III 310C, is usually applied to qualify clean rooms and is a laser-based particle counter. The device suctions air with particles that pass through a laser light. A photodetector detects the loss of the laser light by the scattering effect of the particles. The loss of the laser light allows for determining the obstructing particle’s size. The Lasair III 310C distinguishes the counted particles into six size ranges: 0.3–0.5 µm, 0.5–1 µm, 1–5 µm, 5–10 µm, 10–25 µm, and 25 µm<. To specify the velocity, the volume flow of the device was set to 30 l/min, which is approximately the average breathing volume of humans in calm conditions [28,29]. The particle concentration can vary between 1 and 5 × 10^7^/m^3^ in our laboratory, depending on the weather. According to our validation measurements, in the given range, the particle concentration does not affect the measurement results [28].

The measurement contained two steps. First, the ambient air’s Particle Number Concentration (PNC) [pcs./m^3^] was measured for 1 min. Then, the sample holder with the given mask was connected to the particle counter, and the PNC behind the mask was measured for 1 min. The Particle Filtration Efficiencies (PFEs) for each particle range were calculated from the measured PNC ratios [28]:

(1) $$\mathrm{PFE}=\left(\frac{{\mathrm{PNC}}_{A}-{\mathrm{PNC}}_{C}}{{\mathrm{PNC}}_{A}}\right)\times 100\;\left[\%\right]$$

where PNC_C_ is the particle concentration behind the mask [pcs./m^3^] and PNC_A_ is the particle concentration of the ambient air [pcs./m^3^]. The most applied EN 149:2001 standard [30] provides the filtration efficiencies with aerosols with 0.4 and 0.6 µm MMADs (Median Mass Aerodynamic Diameters). The MMAD analysis of the ambient air in the laboratory showed that in the particle range between 0.3 and 5 µm, the MMAD is between 0.34 and 0.76 µm [28], which is very close to the requirements of EN 149:2001.

## 3. Results

**Figure 5:**
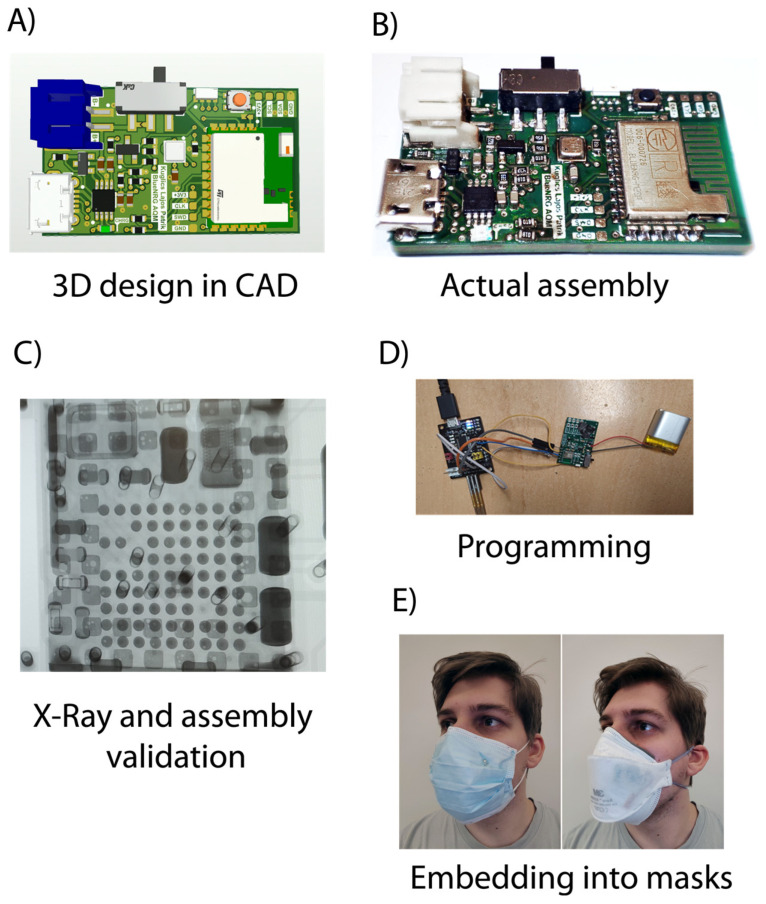
Results of electronics design and assembly. (**A**) 3D design in CAD; (**B**) assembly after reflow soldering; (**C**) X-ray validation of solder joints; (**D**) programming; (**E**) embedding into masks.

The measurement flow was the following: first, the assembly, the consumption, and the final embedding were tested on the modules. The mask filtering capability was then investigated for Types A and B (reference and electronic embedded masks). Then, the sensor data were investigated. The temperature data were read, which revealed the early possibility of respiration analysis. The pressure data evaluation also focused on both the actual pressure values and the possibility of respiration inspection, which was extended by investigating the possible sensor fusion of the two values (pressure and temperature). Then, practical urban use cases were considered and later extended with an analysis of AQI sensor data. Figure 5 shows this flow of work. The data in all presented experiments were forwarded via Bluetooth to a proprietary smartphone application, which enables IoT capability on the go if the phone is connected to the internet.

### 3.1. Consumption and Construction

Construction was deemed successful after programming and X-ray validation. However, current consumption needs further discussion, as presented in Table 1. From the table, the battery requirement can be defined. It was found that a 180 mAh-type battery is sufficient for a use time of 96 h, which is practical for a weekday of work without recharging the battery. This is a proper compromise between applicability and size; the form factor of the battery (36 × 19.5 × 3.9 mm) is almost the same as the PCB. The size of the PCB is 34 × 20 × 4 mm (with components); this shows an able form factor for embedding into the mask.

### 3.2. Results of Filtration Measurements

**Figure 6:**
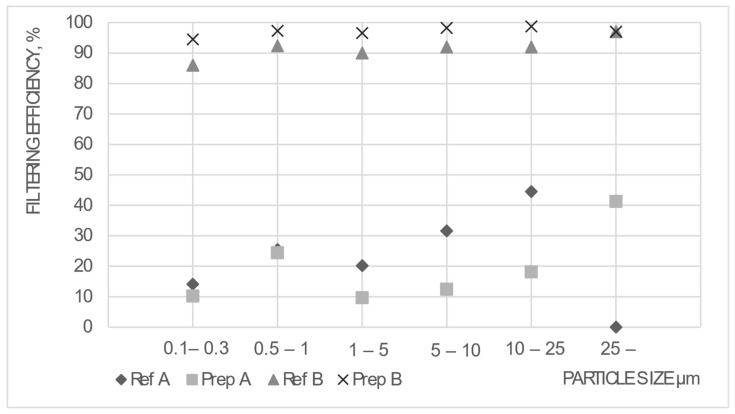
PFE values considering the leakage with Type A and B masks (reference and prepared with the electronic module).

The results of filtration efficiency measurements are presented in Figure 6. The measurement results were separated according to Types A and B: the reference (“Ref” as noted on the figure, unprepared) mask types and the prepared (“Prep” as noted on the figure, with embedded electronic) types. According to Figure 6, it is apparent that the Type A mask (medical) cannot perform according to its filtration capability, even though double layers are used. The electronic embedding further decreases the filtering efficiency. Medical masks generally do not fit well, and any leakage around the perimeter of the mask causes reduced filtering. This is further degraded with the embedding.

The Type B mask is able to filter with high values even after preparation. The professional 3M FFP2 mask fits well on the head model, showing minimal leakage, enabling good performance. The results with the embedded electronics seem even better than those with the reference mask. This may seem contradictory; as a possible explanation, we can say that the reference mask could have a filtering layer that had deviation in its filtering capability. Or, despite careful mask positioning, the embedded electronics caused more considerable tension around the perimeter of the mask, and thus, a better fit to the face.

In conclusion, we can state that embedding did not reduce filtering quality with Type B masks. As such, this type was an optimal target for embedding retrofitted electronics.

### 3.3. Pressure and Temperature Measurements

**Figure 7:**
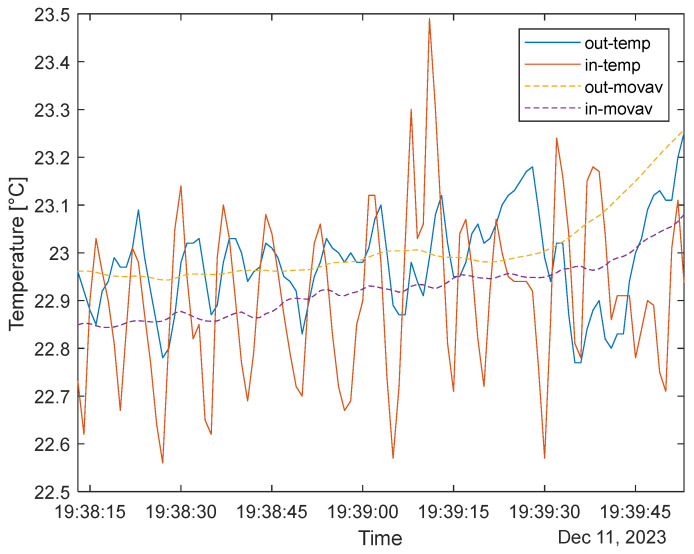
Inside and outside temperature sensor data with (≥1 Hz) data acquisition.

Figure 7 shows the difference between the outside and inside temperature with 10 Hz data acquisition. It can be seen that the outside temperature sensor (out-temp) is lagging behind the inside (in-temp) temperature sensor, as the thermal inertia and the sensor’s placement facing the outside world affect the responsivity. For respiration analysis, the moving-mean methods presented in [31,32] were adapted in the simplest form, without any pre-filtering (10 Hz frequency avoids the use of low pass filters) or optimized moving average window to calculate the respiratory rate. The current paper has no focus on the backend of signal processing nor medical-grade analysis; it investigates the application possibilities, with comparisons between the rise and the fall of the signals, using the moving mean as a threshold.

It can be seen that with the inside sensor, a ~9 respirations/min respiratory rate was recorded, highlighting the dynamics of each breath. This suggests that the user was in regular activity during the measurement. During normal activity, this rate is between 12 and 20 respirations/min [29]; this value is also realistic. The outside sensor performed with an ~8 respirations/min value; however, smaller peak-to-peak values and seemingly more unstable signals mean that data are prone to minor changes in the ambiance. The peak-to-peak value of temperatures stays within 1 °C, and the average moves around with an order of magnitude smaller value in the given time range (below 0.1 °C/min range). Again, it must be emphasized that the current (and later presented) results are not in line with the standards of medical tools and only refer to data prepared for further development or personal information without medical grading.

**Figure 8:**
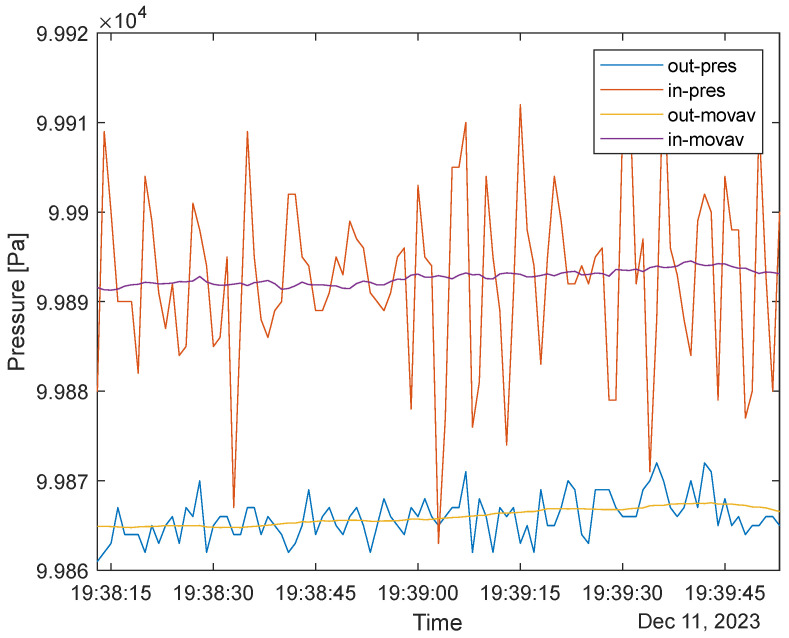
The pressure sensor output difference between mask types; note that the insides have larger peak-to-peak values in the same range.

Figure 8 presents the pressure measurement between the inside and outside of the sensor. It must be noted that the outward-facing sensor signals have much lower peak-to-peak values.

**Figure 9:**
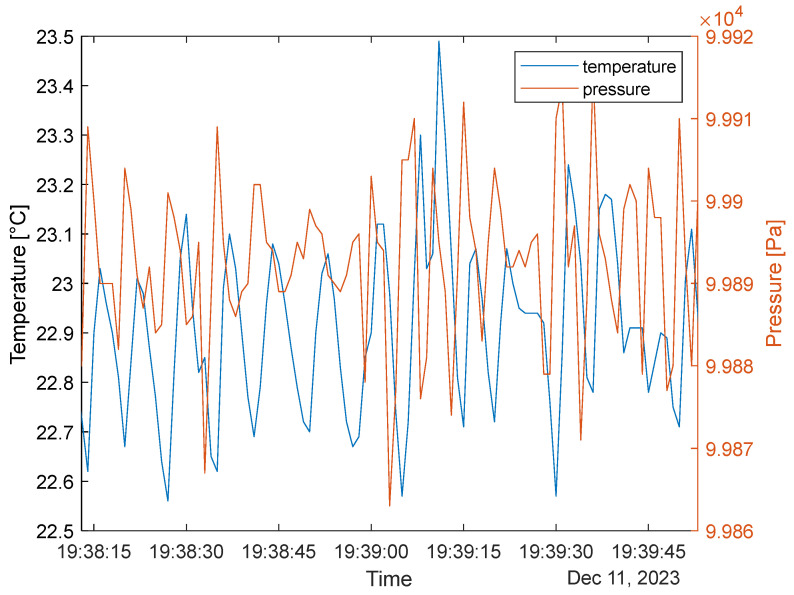
Temperature and pressure sensor measurements in Type B.

The average P_peak-to-peak_IN_ is around 20–40 Pa; the P_peak-to-peak_IN_ average is below 10 Pa. This clearly shows that the outside waveform is a weaker reference for calculating respiration. The recorded rate is 11 respirations/min, similar to findings based on temperature data. However, the larger number comes from more minor changes (at around 19:38:30 and 19:39:00), highlighting minor respiration stutters. At this point, we can confidently assume that inward-facing sensors should be used for user data collection. Figure 9 presents the inward-facing temperature and pressure plot with a Type B mask.

It has to be noted that pressure measurements offer a more responsive recording of breathing due to the device’s thermal inertia. The temperature results are in ~3 s delay in time with the pressure values and align with the pressure characteristics. It must also be noted that eleven peaks were recorded with the pressure sensor in the given time range, while only nine were recorded with the temperature sensor (see previous subsections). This is due to the sensitivity of the pressure sensors, which can be sensitive to various flows [33] inside the mask and minor stutters in the breathing.

### 3.4. Various Use Cases of the System

**Figure 10:**
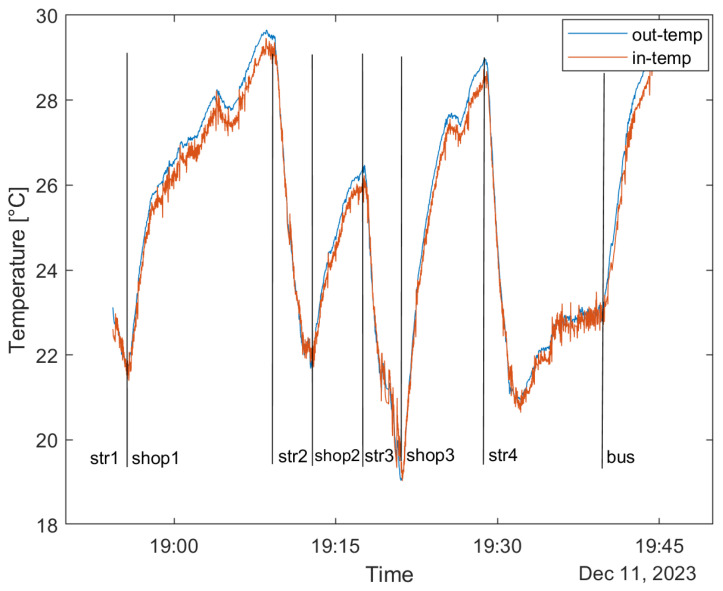
Outer and inside temperature sensor data for approximately an hour of walking in Budapest on 11 December (winter ambiance).

**Figure 11:**
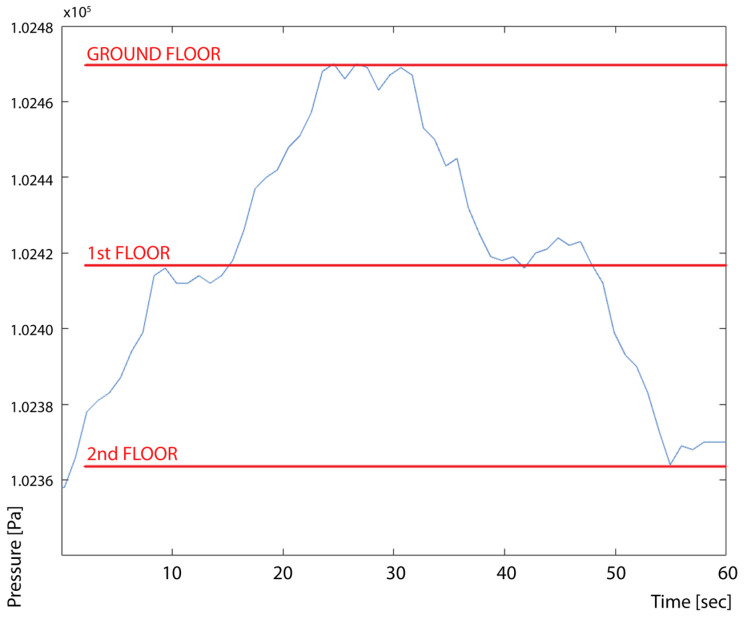
Validation of height according to pressure sensors, urban travel along two stories in an apartment building.

The following scenario is shown in Figure 10. The wearer of Mask Type B was walking in Budapest on 11 December in the evening hours. The outside temperature was around 0 °C. It has to be noted that we are focusing on the large-scale changes, not the respirations (“str” refers to “street”). The figure shows that in such an ambiance, the inside and outside values are practically the same (within Δ1–2 °C), and that temperature does not go under 20 °C for more extended periods. Even after a characteristic change in the signal (shop 3 to street 4), the temperature settles to a ~23 °C temperature. The temperature of the exhalation heats the unit to this temperature. Indoors, the temperature reaches almost 30 °C. Mask Type B is the sturdier construction with better filtering, and it must be noted that the responsivity thus has to be lower; this is the worst case between the two masks, and applicability was found to be satisfactory from this aspect. This method’s absolute temperature measurement is unachievable; however, the system is sensitive to the characteristic changes in the urban scenery. Figure 11 presents a use case where the height is recorded according to the pressure measurement in a building with two stories.

The discussion of the altimetry is as follows. The air pressure at sea level is considered to be 101,325 Pa. The ground floor measurements were recorded at 102,480 Pa—the first floor at 102,420 Pa, the second floor at 102,365 Pa. This means that the values have an offset, which means altitude reading should be oriented with a calibration (the device was unavailable during the studies); however, there was a recorded 55–60 Pa difference found per floor. This shows a 4.5–5 m story height, which aligns with story design standards in Budapest apartment houses (4–5.8 m recorded story heights) [34,35]. The building has an approximately 4.5–5 m story height, meaning the system can follow height changes in urban environments with a 10% maximum error. This is not enough for precise altitude location, but adequate for practical use in everyday life. The BME680 alone could perform with ±0.25% (equivalent to 1 m at 400 m height change) sensitivity; we assume that the uncertainty and offset come from mask embedding and the thermal effect on the sensor and its calibration procedures.

### 3.5. Discussion on Temperature-Pressure Fusion Possibilities

According to the previous results, the fused data may be used for respiration analysis. Summarizing findings on this aspect, we can say that the temperature enables better respiration-rate monitoring due to its insensitivity to minor stutters in respiration or turbulent flows inside the masks. However, large changes in ambient temperature (such as during a winter walk, on a hot day, or with air conditioning inside) practically exclude the possibility of temperature-based respiration-rate analysis. So, the following is suggested for fusion. In steady temperature averages, the temperature should be used as the basis for respiration rate; however, if the average of the temperature is diverted from the steady state (by > 0.1 °C/min; see Section 3.3 presenting below 0.1 °C/min moving average), the pressure sensor should be used, which is insensitive in this matter to temperature changes.

To discuss this point, we refer to the datasheet [20]: the temperature coefficient offset is ±1.3 Pa/K; this is sufficient, as the inner pressure sensor’s peak-to-peak value is situated in the ~10–100 Pa range (see Section 3.4). The pressure changes per story (~50 Pa in ~10 s range in, e.g., urban elevation) will not interfere with the given ~10–50 Pa/s dynamics during respiration. It is unlikely that a situation where significant temperature and pressure average changes will occur; however, if that happens, the system should pause providing feedback and highlight that it is waiting for a state where processing could restart. The aforementioned respiration filtering optimizations [31] could be adapted to improve these aspects.

### 3.6. Discussion on Humidity

**Figure 12:**
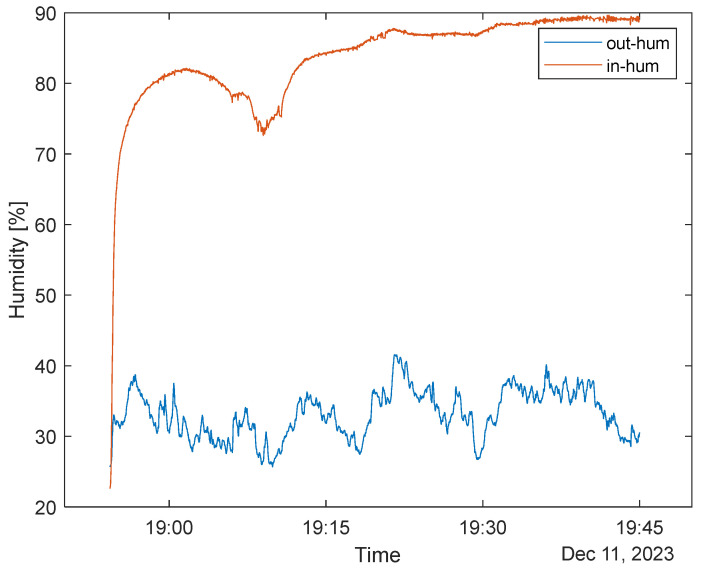
Humidity sensor measurements (outside–inside mask).

Figure 12 presents the humidity measurements of the inner and outer sensors in the Type B mask. It must be noted that inner humidity nearly saturates the inner sensor after 20–30 min, meaning no valuable information can be obtained from this unit. The outside sensor measured data between 30–40% values. The weather outside was exceptionally humid during the measurement in Budapest in 2023 (RH% between 80–100%, [36]); this means that the humidity analysis was imprecise for further analysis on both sides.

### 3.7. AQI Analysis

**Figure 13:**
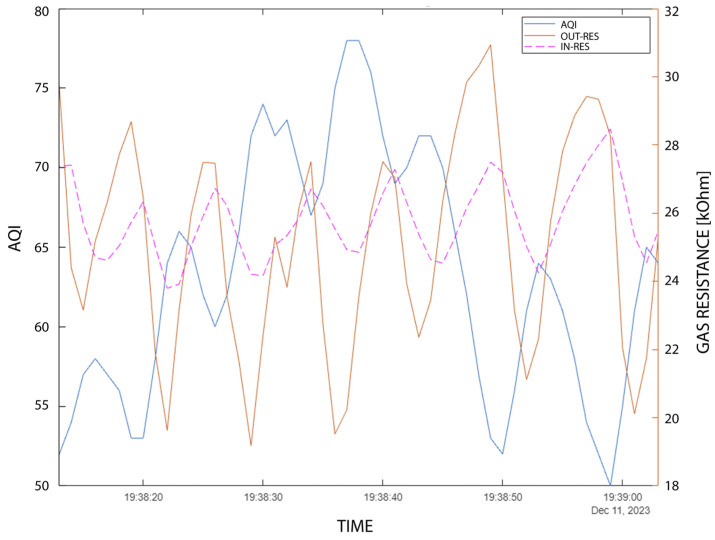
AQI and gas sensor resistance measurement.

Figure 13 presents the analysis of gas sensor resistance and the resulting AQI after the BSEC calibration [20,22]. The situation in respiration analysis is similar to the cases presented in Section 3.3; however, it is interesting that the peak-to-peak is more stable in the case of the outer sensor (~Δ10 kOhm) than in the case of the inner sensor (~Δ3 kOhm). This is because the inner sensor is more prone to saturation.

AQI numbers are not suggested for use in respiration calculations. The variation of the AQI is between 50 and 80 in the given time window (~minute) with a 10–20 AQI amplitude. According to the datasheet [20], the AQI classes are the following: 0–50 (excellent); 51–100 (good); 101–150 (lightly polluted); 151–200 (moderately polluted); 201–250 (heavily polluted); 205–350 (severely polluted); and >351 (extremely polluted). This means that the measurement took place in a “Good” environment, and the measurements stayed within the AQI class range.

**Figure 14:**
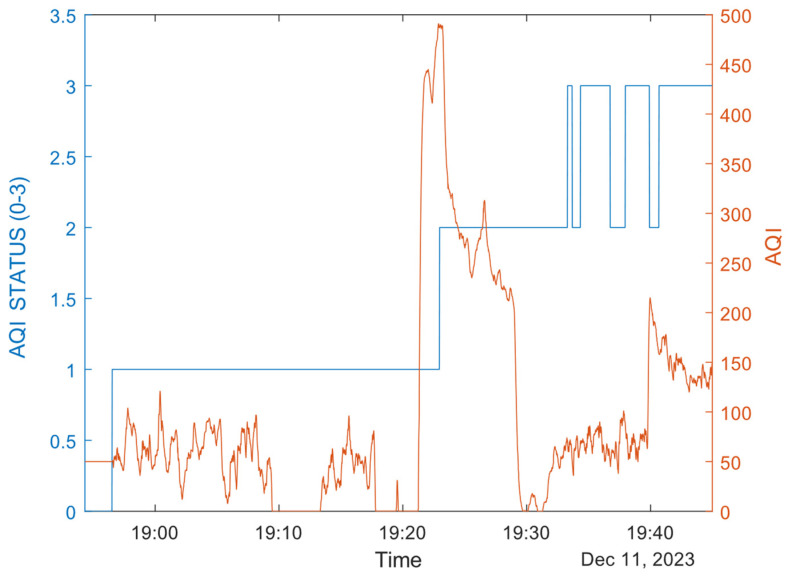
AQI measurement and AQI precision status (0–3 state).

The library has four levels of AQI accuracy: 0—stabilization in progress, 1—calibration required, 2—calibration in progress, and 3—calibrated. Over time and with frequent changes in ambient air pollution, the algorithm can determine air quality with increasing accuracy. As can be seen, the boundary between outdoor and indoor air is very well separated, as fresh air outside always brings the AQI value down to 0. This is shown in Figure 14. Compared with Figure 10 (as these two urban scenarios are practically the same), it can be seen that the AQI was heavily degraded during “SHOP3” attendance (between 19:20 and 19:30) and was also increased during the “BUS” trip (AQI 150–200, after 19:40). The street measurements (after the AQI status changed to 2–3, after 19:25) show values between 50 and 100. Before that, the AQI measurements are unreliable. The system requires more than half an hour to set itself to a calibrated AQI measurement. So, AQI detection is only suggested when the mask is used for a while, which is a limitation of our system coming from the sensor itself.

## 4. Conclusions

In this paper, we realized a conceptual smart sensor node with IoT capabilities embedded into personal protective face masks and evaluated it in urban environments. The novelty comes from the form factor, the multi-parameter measurement approach, and the inside–outside sensor positioning, which simultaneously enables personal monitoring and outside-world evaluation, with possible fusion options. The setup uses standard protective materials and design, with real-time IoT communication capabilities, a working application, prospects of background data processing, and alignment with future trends, according to [37,38]. We optimized the form factor (36 × 20 × 4 mm PCB, and almost the same as the battery) to be embedded between two medical masks or into a professional, e.g., 3M Aura 9320D + FFP2 type personal protective mask. The device can communicate with a smartphone app, extending IoT usability. The battery life ensures the device can work for a working week (more than 90 h). It was found that the medical masks have a weak fit on the face, so filtration (essential protective function) fails with the embedding. The FFP2 professional mask could withstand the embedding process, where the filtering efficiency practically remained in a similar range.

We showed that the temperature sensor can follow the outside and inside temperatures; however, absolute temperature measurement is not possible due to exhalation, which heats the sensors. The temperature of the inner and outer sensors has a minimal (~±1 °C) difference. The inner temperature sensor can detect respiration with 10 Hz data acquisition using a simple moving-mean method. While thermal inertia smooths out temperature recordings, pressure measurement is more sensitive to turbulent flows inside the mask or slight stutters in respiration. As a fusion, it is suggested that the inner temperature should be used as the basis for the respiration rate; however, if the average temperature is rapidly changing (moving out of the steady state) by >0.1 °C/min, the inner pressure sensor should be used, which is insensitive in this matter. Also, the outer pressure sensor can be used as an altimeter in an urban environment, highlighting dynamic changes in the surroundings, such as rising/descending between stories, with 10% precision. The humidity of the system is saturated within the range of 10 min. “Outside” humidity values are also unreliable and altered by the filtering nature of the mask, which captures humidity. AQI analysis is not applicable to inspecting the respiration rate; however, after half an hour of settling, the outer sensor can capture the AQI defined by the manufacturer of the sensor. We showed use cases in various urban environments. AQI analysis is suggested if the user is wearing the device for longer (e.g., personal protective equipment during hours of work).

The results promote smart city concepts and help endeavors related to SDGs (Sustainable Development Goals [39]) 3 and 11, regarding good health and clean, sustainable cities and urban environments. Future development has different potential paths. Work will continue with the backend development from data to improve respiration detection capabilities. Further use cases could help validate various data from the working environment. Also, the involvement of textile masks [28,40] and the improvement of the libraries and future sensors (e.g., a 688-type update on the applied units) will enable faster setting to a work point in the AQI measurements, more reliable use, and possible AI enhancements.

## Figures and Tables

**Table 1:** Table of current consumption.

Components	Time Ratio [Sec]	Current [mA]	Consumption [mAsec]
2× BME680	1	1.8	1.8
DC-DC converter	1	0.06	0.06
MCU low-power mode	0.9995	2.1 × 10^−3^	2.1 × 10^−3^
MCU BLE 2dBm	1.6 × 10^−4^	17.2	2.75 × 10^−3^
MCU I2C transmittance	3.36 × 10^−4^	1	3.36 × 10^−4^
FET leakage	1	0.001	0.001
MCU ADC measurement	1.10 × 10^−8^	2	2.2 × 10^−8^
V divider + ADC meas. curr.	1.10 × 10^−8^	2.25	2.47 × 10^−8^
		**Summary:**	**1.8662**

## Data Availability

Data is available upon request.
